# The cumulative effect of core lifestyle behaviours on the prevalence of hypertension and dyslipidemia

**DOI:** 10.1186/1471-2458-8-210

**Published:** 2008-06-13

**Authors:** Raquel Villegas, Patricia M Kearney, Ivan J Perry

**Affiliations:** 1 Department of Epidemiology and Public Health, University College Cork, Cork, Ireland

## Abstract

**Background:**

Most cardiovascular disease (CVD) occurs in the presence of traditional risk factors, including hypertension and dyslipidemia, and these in turn are influenced by behavioural factors such as diet and lifestyle. Previous research has identified a group at low risk of CVD based on a cluster of inter-related factors: body mass index (BMI) < 25 Kg/m^2^, moderate exercise, alcohol intake, non-smoking and a favourable dietary pattern. The objective of this study was to determine whether these factors are associated with a reduced prevalence of hypertension and dyslipidemia in an Irish adult population.

**Methods:**

The study was a cross-sectional survey of 1018 men and women sampled from 17 general practices. Participants completed health, lifestyle and food frequency questionnaires and provided fasting blood samples for analysis of glucose and insulin. We defined a low risk group based on the following protective factors: BMI <25 kg/m^2^; waist-hip ratio (WHR) <0.85 for women and <0.90 for men; never smoking status; participants with medium to high levels of physical activity; light alcohol consumption (3.5–7 units of alcohol/week) and a "prudent" diet. Dietary patterns were assessed by cluster analysis.

**Results:**

We found strong significant inverse associations between the number of protective factors and systolic blood pressure, diastolic blood pressure and dyslipidemia. The prevalence odds ratio of hypertension in persons with 1, 2, 3, ≥ 4 protective factors relative to those with none, were 1.0, 0.76, 0.68 and 0.34 (trend p < 0.01). The prevalence odds ratio of dyslipidemia in persons with 1, 2, 3, ≥ 4 protective factors relative to those with none were 0.83, 0.98, 0.49 and 0.24 (trend p = 0.001).

**Conclusion:**

Our findings of a strong inverse association between low risk behaviours and two of the traditional risk factors for CVD highlight the importance of 'the causes of the causes' and the potential for behaviour modification in CVD prevention at a population level.

## Background

CVD is a leading cause of morbidity and mortality worldwide [1]. Hypertension and dyslipidemia are two of the major traditional risk factors for CVD [2-4]. While it has previously been reported that only 50% of CVD can be attributed to traditional risk factors [5,6], it is now recognized that these factors occur much more frequently in people with cardiovascular disease [7,8]. It is well established that treatment of hypertension and dyslipidemia results in a reduction in CVD [9,10]. The effect of behavioural factors such as diet and lifestyle is less clear. These behavioural factors may be powerful predictors of hypertension and dyslipidemia and interventions to influence these behavioural factors have the potential to have a major impact on CVD. In particular, while treatment of hypertension and dyslipidemia requires diagnosis and treatment of individual patients, behavioural factors are amenable to population level interventions. The Nurses' health study identified a cluster of protective behaviours that predicted low risk of cardiovascular disease and diabetes [11,12]. In a previous study we have shown that a cluster of inter-related factors (BMI, WHR, never smoking status, medium to high levels of physical activity, light alcohol consumption and a prudent diet) are associated with a significantly reduced risk of glucose intolerance and insulin resistance [13]. In this latter paper we have suggested that work on chronic non-communicable disease such as CVD needs to address the clustering and inter-relatedness of major dietary and lifestyle factors. The aim of the study was to investigate the association between these previously identified protective behavioural factors and the prevalence of hypertension and dyslipidemia in an Irish adult population.

## Methods

### Design, subjects and methods of data collection

The Cork and Kerry Diabetes and Heart Disease Study is a cross-sectional survey of the prevalence of glucose intolerance and associated CVD risk factors in an Irish adult general population sample. Details of the methods of data collection, including the self-completed questionnaire and physical measurements (height, weight, waist and hip circumference) and fasting blood samples have been reported previously [14]. Stratified random sampling by age and sex was utilized to recruit equal numbers of men and women in four age strata between the ages of 50 and 69 years. Subjects with CVD, diabetes mellitus or other chronic disease, and those receiving medication were included in the sample base. From a total of 1473 men and women from 17 general practice lists in the South of Ireland who were invited to participate, 1018 attended for the assessment, (491 men and 527 women), resulting in a response rate of 69.1%.

The Ethics Committee of the Cork Teaching Hospitals approved the study protocol. All participants gave signed informed consent, including consent to use their data for research purposes.

### Questionnaires and physical measurements

#### Physical activity

Physical activity was assessed using the British Regional Heart Study questionnaire [15]. This questionnaire addresses the type and duration of exercise while travelling to work; participants' assessment of their occupational physical activity; grading (1–5) of weekend physical activity; frequency of participation in active physical exercise such as running, digging, and tennis; and the number of years the subject had been involved in such activity. A physical activity score was derived based on the frequency and intensity of the activities reported, using recommendations of a National Heart, Lung, and Blood Institute workshop and the Minnesota intensity codes [15]. The use of this physical activity score has been validated previously against heart rate and forced expiratory volume in 1 second in men free of pre-existing coronary heart disease [15]. Participants were grouped into three physical activity categories based on their physical activity scores: inactive & occasionally active (*Low*, n = 407), light to moderate activity (*Medium*, n = 387) and moderate to vigorous activity (*High*, n = 144). Data on physical activity were not available for 80 participants.

#### Smoking status

Participants were classified according to their current smoking status into one of three categories: never smoker (n = 463), ex-smoker (n = 341) and current smoker (n = 190). Data on smoking were not available for 33 participants. Never smokers were defined as those who answered no to the questions: "Do you regularly smoke cigarettes at present?" and "if no, have you ever regularly done so?". Those who smoked a pipe or cigars (n = 2) and former cigarette smokers who smoked a pipe or cigars (n = 11) were regarded as current smokers.

#### Socio-economic status

Participants were classified into five socio-economic status (SES) categories based on a combination of the Irish Central Statistics Office (CSO) classification system and data on educational attainment. Category I consisted of participants classified by the CSO as higher and lower professionals, and employers/managers and own account workers with third level education (n = 161). Category II consisted of participants who were employers, manager or own account workers and did not have third level education (n = 64). Category III consisted of self employed farmers (n = 138). Category IV consisted of non-manual workers and skilled and semi-skilled manual workers (n = 371). Category V consisted of all agricultural workers and non-skilled manual workers (n = 255). Information on SES was not available for 29 participants. When a participant defined herself as a housewife, the occupation of her partner was used for classification purposes.

#### Dietary data

We used a food frequency questionnaire (FFQ) adapted from the UK-EPIC study instrument [16] and subsequently modified by the Irish National Nutritional Surveillance Unit to reflect the Irish diet [15-17]. The FFQ has been validated in an Irish adult population by comparison with seven day weighed records and the use of one biomarker, 24-hour urinary nitrogen [17-19]. Of the 1018 participants, 937 (92%) completed the FFQ. We excluded participants that had implausible scores for total energy intake (<500 or > 3500 kcals/day for women and <800 or >4200 kcal/day for men), [18] leaving 851 (84%) participants with dietary data for the analysis.

#### Alcohol intake

Alcohol intake was estimated primarily from the FFQ data. Alcohol intake (ethanol) was originally estimated in grams per day, using the McCance and Widdowson's Food composition tables, [20] and was subsequently converted into units per day, assuming an average of one unit per 12 g of ethanol [21]. Alcohol consumption data from the FFQ (categorized in units per day) were cross-checked with the data from the lifestyle questionnaire. Inconsistent reports from the two questionnaires were coded as unclassified. Participants were classified into seven categories according to their alcohol intake: never (n = 281), occasional (<0.5 units/day; n = 341), light (0.5–0.99 units per day; n = 118), moderate (1.0–2.99 units per day; n = 70), heavy (>3 units per day; n = 49), ex-drinkers (n = 43) and unclassified (n = 31). Data were not available for 85 participants.

#### Determination of dietary patterns by K cluster analysis

Dietary patterns were determined by K-means cluster analysis, using MINITAB software programme (version 13), [22-24]. We based the cluster analysis on food variables. Items from the FFQ were expressed in terms of the proportion of total mass of food consumed (g/day) or mls/day in the case of alcoholic drinks or beverages. These were subsequently aggregated into 22 mutually exclusive food groups similar to those used by Pryer and colleagues [24], which were based on the 51 food/drinks groups defined by Gregory and co-workers [25]. Continuous food groups were standardised by converting them to standard normal deviates to ensure that clusters were not influenced by food groups with high specific gravity (such as beverages). We identified the number of clusters and seeds by conducting principal component analysis with the food groups [26]. Three dietary clusters were identified, a 'traditional diet', an 'alcohol and convenience food diet' and a 'prudent diet' cluster. The 'prudent diet' group had the highest intake of pasta and rice, brown breads and unrefined cereals, spreads, poultry, fish, low fat milk and dairy products, salad dressing, fruit and vegetables and the lowest intake of chips, white bread and refined cereals, butter, high fat dairy, meat, meat products and sweets. This prudent dietary pattern represents a favourable nutrient profile, characterised by a higher intake of polyunsaturated fat, antioxidant vitamins and fibre and a lower intake of saturated fat (27).

#### Anthropometric measurements

BMI was used as an index of relative weight. Waist and hip measurements were taken using standard methods [9], and WHR was used as a measure of central obesity. The data on height, weight, waist and hip circumference were based on the mean of two measurements. Blood samples were taken for fasting lipids. Lipoprotein profiles were analysed using the Roche Hitachi 747 Multichemistry analyser and the Olympus 640 Discrete analyser. For analysis of the fasting lipids, we excluded participants who did not fast for more than 8 hours (n = 51) or did not provide information on their fasting status (n = 50) and one participant with type 1 diabetes mellitus. Following exclusions, data on fasting triglycerides and high density lipoprotein (HDL) cholesterol were available for 914 and 901 participants respectively. Non-fasting and unknown fasting status participants were similar in age, gender and socio-economic status to those with known fasting status. Blood pressure was measured under controlled conditions by a trained research nurse using a portable digital blood pressure monitor (Omron HEM-705CP). Blood pressure was measured three times in succession with the subject seated, with left arm at heart level, and cuff adjusted for arm circumference. For each participant, the mean of the second and third readings was used in analysis of systolic blood pressure (SBP) and diastolic blood pressure (DBP) data. Data on measurements of blood pressure were available for 1016 participants.

#### Definition of hypertension

Hypertension was defined as SBP ≥ 140 mm Hg and/or DBP ≥ 90 mm Hg and/or use of anti-hypertensive medication.

#### Definition of dyslipidemia

Dyslipidemia was defined as triglycerides ≥ 1.7 mmol/L and/or HDL cholesterol <0.9 mmol/L in men and <1.0 mmol/L in women.

#### Definition of pre-existing cardiovascular disease

Pre-existing CVD was determined based on the following: a self reported history of myocardial infarction or angina or a history of coronary artery bypass graft or coronary artery angioplasty or a positive 'Rose Questionnaire' or a history of stroke, peripheral vascular disease or abdominal aortic aneurysm or evidence of a definite previous myocardial infarction on an analysis of the study electrocardiographs by a single experienced cardiologist [14].

#### Low risk group criteria

Six protective factors were defined: healthy body weight (BMI ≤ 25 kg/m^2^); absence of central obesity (WHR <0.90 for men and WHR <0.85 for women); physically active (combination of medium and high activity groups); never smokers; light drinkers (3.5–7 units of alcohol per week) and a prudent dietary pattern.

#### Statistical analysis

Analysis of variance and covariance was used to determine the differences in means of SBP, DBP, fasting triglycerides and HDL cholesterol in individuals with different numbers of protective factors. In these analyses, we used the natural log of triglycerides as the dependent variable to correct for skewness. Logistic regression analysis was performed with hypertension and dyslipidemia as the dependent variables and the number of protective factors as the independent variable. Participants with 4, 5 or 6 protective factors were combined into a group of 4 or more factors for the analyses. Odds ratios and 95% confidence intervals were calculated for each category (having 1, 2, 3, 4 or more protective factors) relative to those with none of the protective factors. Analyses were adjusted by age, sex, SES and history of pre-existing CVD. We also adjusted for kcals/day, as hypertension and dyslipidemia may be associated with total energy intake [27].

## Results

The prevalence of hypertension and dyslipidemia and each of the six protective factors was similar in all participants and the subset of participants with data on all variables (Table 1). The distribution of the number of protective factors among those with valid data on all risk factors is shown in Table 2. Overall 7.5% (95% C.I. 5.8–9.7) of the sample had no protective factors; 12.5% (95% C.I., 9.3–16.4) of men vs. 3.0% (95% C.I. 1.6–5.2) of women, p < 0.001. Only 5.3% (95% C.I. 3.2–8.1) of men had four or more protective factors as compared with 20.6% (95% C.I.16.6–25.0) of women, p < 0.0001. We found no significant association between the number of low risk factors and SES.

**Table 1:** Prevalence of hypertension, dyslipidemia and each of the protective factors among all participants and subset with valid data on all variables

**Risk Factor**	**All Participants (N = 1018)**	**Subset (N = 759)**
Hypertension	47.2%	43.3%
Dyslipidemia	29.0%	29.7%
BMI ≤ 25 kg/m^2^	27.5%	27.3%
WHR<0.85 women, <0.90 men	26.9%	27.9%
Medium and high level of physical activity	56.6%	57.0%
Light drinkers	12.7%	13.0%
Never smokers	46.6%	48.0%
Healthy eating cluster	39.8%	40.3%

**Table 2:** Distribution of protective factors among subset of participants with valid data on all variables

**Number of Protective Factors**	**N**	**Percentage**
No Factors	57	7.5%
One Factor	189	24.9%
Two Factors	235	31.0%
Three Factors	177	23.3%
Four Factors	76	10.0%
Five Factors	23	3.0%
Six Factors	2	0.3%

**Figure 1:**
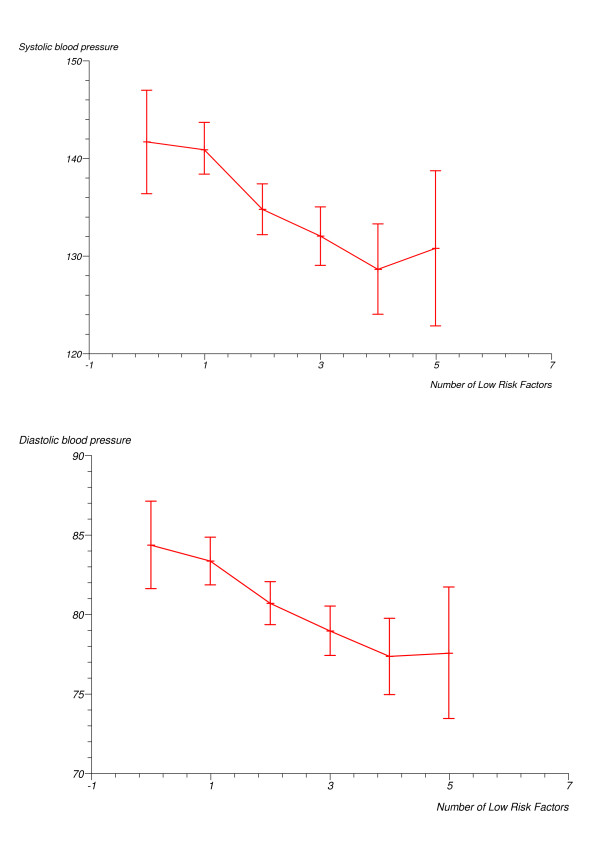
Mean systolic and diastolic blood pressure (mmHg) by the number of protective factors (5 and 6 factors combined).

**Figure 2:**
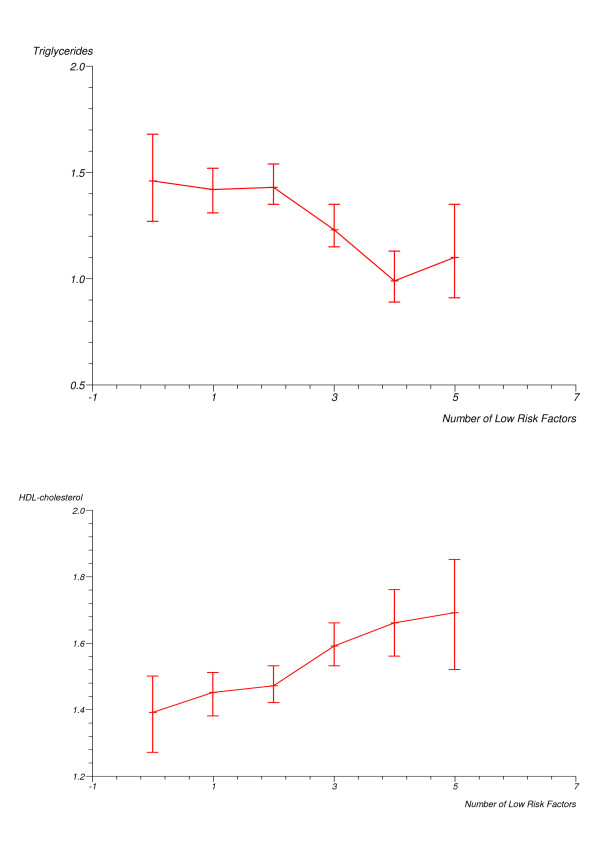
Means of blood lipids (mmol/L) by the number of protective factors (5 and 6 factors combined).

Associations between individual protective factors and the prevalence of hypertension and dyslipidemia are shown in Table 3. We found significant inverse associations between the individual behavioural factors low BMI and low WHR and the prevalence of hypertension. Low BMI, low WHR and never smoker status were inversely and significantly associated with the prevalence of dyslipidemia. There were strong and highly significant inverse associations between mean SBP, mean DBP, and triglycerides and the number of protective factors, and a strong highly significant direct association between the number of factors and HDL cholesterol (Figures 1 and 2). There was an inverse relationship between the number of protective factors and the prevalence of hypertension with a significant trend for a reduction in hypertension prevalence with increasing number of protective factors (Table 4). Similar significant linear inverse trends were observed in analyses excluding participants with previously diagnosed type 2 diabetes. The prevalence odds ratios in fully adjusted analyses following exclusion of previously diagnosed diabetics were 0.83, 0.98, 0.49 and 0.24, (p for trend = 0.001), in persons with 1, 2, 3, ≥ 4 low risk factors respectively, relative to those with none. Similarly, there was a significant linear inverse trend in the prevalence of dyslipidemia with increasing number of low risk factors (Table 5). This was observed in age and sex adjusted analyses, as well as in fully adjusted analyses. The prevalence odds ratios of dyslipidemia in fully adjusted analyses after exclusion of those with previously diagnosed diabetes were 1.00, 0.76, 0.68 and 0.34, in persons with 1, 2, 3, ≥ 4 low risk factors respectively, relative to those with none (p for trend < 0.01).

**Table 3:** Associations* between individual protective factors and the prevalence of hypertension and dyslipidemia

	**OR**	**95% CI**	**P-value**
**Hypertension**			
Low BMI	0.40	0.28–0.59	<0.001
Low WHR	0.60	0.40–0.59	0.01
Physically active	1.09	0.79–1.50	0.59
Never smoker	0.77	0.56–1.06	0.11
Light drinking	1.07	0.77–1.47	0.69
Healthy eating cluster	1.10	0.70–1.74	0.67
			
**Dyslipidemia**			
Low BMI	0.32	0.19–0.54	<0.01
Low WHR	0.48	0.29–0.80	<0.01
Physically active	1.15	0.79–1.67	0.87
Never smoker	0.58	0.40–0.85	<0.01
Light drinking	1.15	0.79–1.67	0.46
Healthy eating cluster	1.10	0.65–1.89	0.70

* Adjusted for age, sex, SES, pre-existing CVD and Kcals/day and for each other

**Table 4:** Hypertension prevalence odds ratio by the number of protective factors

**Number of Protective Factors**	N*	**OR+**	**(95% CI)**	**P-value Trend<0.001**	**OR++**	**(95% CI)**	**P-value Trend<0.01**
**0**	57	1.00			1.00		
**1**	189	0.98	0.54–1.80	0.95	1.00	0.55–1.89	0.95
**2**	235	0.73	0.40–1.33	0.31	0.76	0.42–1.40	0.39
**3**	177	0.62	0.33–1.16	0.14	0.68	0.36–1.27	0.22
**≥4**	101	0.31	0.15–0.62	0.001	0.34	0.16–0.70	<0.01

*N will vary because of missing data

+Adjusted for age and sex

++Adjusted for age, sex, SES, pre-existing CVD and Kcals/day

**Table 5:** Dyslipidemia prevalence odds ratio by the number of protective factors

**Number of Protective Factors**	N*	**OR**	**(95% CI)**	**P-value Trend<0.001**	**OR++**	**(95% CI)**	**P-value Trend = 0.001**
**0**	51	1.00			1.00		
**1**	161	0.78	0.40–1.50	0.45	0.83	0.42–1.62	0.58
**2**	208	0.96	0.51–1.82	0.90	0.98	0.51–1.90	0.96
**3**	162	0.51	0.26–1.01	0.05	0.49	0.24–1.00	0.05
**≥4**	92	0.23	0.09–0.55	0.001	0.24	0.09–0.59	<0.001

*N will vary because of missing data

+Adjusted for age and sex

++Adjusted for age, sex and SES, pre-existing CVD and Kcals/day

## Discussion

In this cross-sectional study of middle-aged Irish men and women, a combination of 6 core protective factors (normal BMI, WHR below the current threshold for central obesity, never smoking, light alcohol consumption, a prudent diet and regular physical activity) was associated with a significantly lower prevalence of hypertension and dyslipidemia. There was a strong trend for a reduced prevalence of hypertension and dyslipidemia with increasing number of protective factors with those who scored four or more factors having approximately one-third and one-quarter the risk of hypertension and dyslipidemia respectively.

However, this study had limited power to detect associations between individual protective factors and the prevalence of hypertension and dyslipidemia and indeed only the anthropometric measurements were significantly associated with the prevalence of both hypertension and dyslipidemia while never smoker status was also significantly associated with dyslipidemia. Another limitation of our study is the cross-sectional design. This design prohibits any inferences of a causal association between the protective factors and hypertension and dyslipidemia and in addition prevents us from determining the direction of the association between protective lifestyle behaviours and hypertension and dyslipidemia. It may be that individuals with high blood pressure or an abnormal lipid profile are less likely to adopt healthy lifestyles rather than the lifestyles having a protecting effect against the development of hypertension and dyslipidemia. Despite these limitations, our study extends and compliments the findings from major longitudinal studies in men and women [28-31] which have reported on the combined impact of protective lifestyle factors and morbidity and mortality and suggests that the effects of these factors may be mediated by an effect on hypertension and dyslipidemia.

To date much of the focus in CVD prevention and health promotion has been on determining the individual contribution of risk factors to the development of cardiovascular disease. However it is now accepted that virtually all of the population attributable risk of myocardial infarction is due to well studied factors such as smoking, hypercholesterolemia, diabetes mellitus, hypertension and obesity [32]. Moreover, while the independent contribution of individual risk factors is interesting from an etiological perspective, in terms of disease prevention and public health it is less critical. More important than quantifying the magnitude of risk conferred by the addition of each individual factor is the effect of these factors in combination and the consequent potential for prevention in terms of lifestyle changes that address these factors simultaneously.

## Conclusion

To date much research funding and resources have been expended in elucidating the causal role of the risk factors for CVD. However, it is now evident from a health policy perspective that interventions at a population level addressing diet, exercise and smoking, will have profound effects on blood pressure and lipid levels in the population and ultimately on the incidence of cardiovascular disease. Thus the challenge for public health is to align public policy and health systems towards increasing the population prevalence of core protective factors for CVD.

## Abbreviations

BMI: Body Mass Index; CSO: Central Statistics Office; CVD: Cardiovascular Disease; DBP: Diastolic Blood Pressure; FFQ: Food Frequency Questionnaire; HDL: High Density Lipoprotein; SBP: Systolic Blood Pressure; SES: Socio-Economic Status; WHR: Waist-Hip Ratio.

## Competing interests

The authors declare that they have no competing interests.

## Authors' contributions

RV completed data analysis and worked on early drafts of the manuscript. PMK edited the manuscript and approved the final version for publication. IJP designed and conducted the Cork and Kerry study, edited the manuscript and approved the final version for publication. All authors have read and approved the final manuscript.

## Pre-publication history

The pre-publication history for this paper can be accessed here:


